# Oral antibiotic prophylaxis for infection in patients with vascular anomalies receiving sirolimus treatment: a multicenter retrospective study

**DOI:** 10.1186/s13023-023-02740-3

**Published:** 2023-05-24

**Authors:** Tong Qiu, Yanan Li, Xue Gong, Jiangyuan Zhou, Kaiying Yang, Xuepeng Zhang, Zixin Zhang, Yuru Lan, Fan Hu, Qiang Peng, Yongbo Zhang, Feiteng Kong, Siyuan Chen, Yi Ji

**Affiliations:** 1 Division of Oncology, Department of Pediatric Surgery, West China Hospital of Sichuan University, Chengdu, 610041 China; 2 Med-X Center for Informatics, Sichuan University, Chengdu, 610041 China; 3 Department of Vascular and Interventional Radiology, West China Second University Hospital, Sichuan University, Chengdu, 610041 China; 4 Department of Pediatric Surgery, Chengdu Women and Children’s Central Hospital, Chengdu, 610031 China; 5 Department of Pediatric Surgery, Sichuan Provincial Maternity and Child Health Care Hospital, Chengdu, 610045 China; 6 Pediatric Intensive Care Unit, Department of Critical Care Medicine, West China Hospital of Sichuan University, 37# Guo-Xue-Xiang, Chengdu, 610041 China

**Keywords:** Vascular anomalies, Sirolimus, Infection, Antibiotic prophylaxis, Trimethoprim, Sulfamethoxazole

## Abstract

**Objectives:**

Patients with vascular anomalies (VAs) who receive oral sirolimus may be at high risk of infectious complications. Antibiotic prophylaxis with trimethoprim-sulfamethoxazole (TMP-SMZ) has been advocated. However, there have been few evidence-based analyses on this topic. This study assessed the effect of prophylactic TMP-SMZ on the incidence of infections in VA patients receiving sirolimus monotherapy.

**Methods:**

A retrospective, multicenter chart review was performed on all VA patients receiving sirolimus treatment from August, 2013 to January, 2021.

**Results:**

Before January 2017, 112 patients were treated with sirolimus without antibiotic prophylaxis. In the subsequent period, 195 patients were treated with TMP-SMZ for at least 12 months during sirolimus therapy. The percentage of patients with at least one serious infection during the initial 12 months of sirolimus treatment did not differ between the groups (difference, 1.1%; 95% CI − 7.0–8.0%). We observed no difference in the incidence of individual infection or total adverse events between the groups. The rate of sirolimus discontinuation due to adverse events did not differ significantly between groups.

**Conclusions:**

We demonstrated that prophylactic TMP-SMZ does not decrease the incidence of infection or improve tolerance in VA patients receiving sirolimus monotherapy.

**Supplementary Information:**

The online version contains supplementary material available at 10.1186/s13023-023-02740-3.

## Introduction

Vascular anomalies (VAs), including vascular tumors and vascular malformation, represent a broad spectrum of diseases from a simple ‘birthmark’ to life-threatening lesions [1]. Growth or expansion of VAs can lead to clinical problems, such as disfigurement, bleeding, recurrent infection, chronic pain, thrombocytopenia, consumptive coagulopathy, organ dysfunction, and death [2–5]. Patients with complicated VAs may suffer from progressive clinical symptoms with decreased quality of life [6]. Because of the broad spectrum and insufficient treatment regimens, the management of complicated VAs is complex and usually needs a multidisciplinary team with a combination of surgical, interventional (e.g., embolization), and medical treatments [7, 8].

In the past decade, sirolimus, which is well known as an immunosuppressive drug to prevent organ rejection, has emerged as a new pharmacologic treatment for VAs. There is mounting evidence that oral sirolimus treatment can improve the prognosis of VAs, most notably kaposiform hemangioendothelioma (KHE) with life-threatening Kasabach-Merritt phenomenon (KMP), venous malformation and lymphatic malformation [9–16]. However, although sirolimus is efficacious, safety is a main issue of concern for treating VAs as many patients need long-term therapy (usually more than 1 year). In addition, most of these patients are of pediatric age. Because the intrinsic immunosuppressive potential of sirolimus, there is a potential high risk for infections. In fact, sirolimus-related infections, such as *Pneumocystis jirovecii* pneumonia (PJP), can be fatal [17, 18]. Even when infections are not fatal, they frequently result in substantial delays and treatment discontinuation, leading to an increased risk of treatment failure. As such, this situation poses a potential requirement for antibiotic prophylaxis in the setting of oral sirolimus treatment. While antibiotic prophylaxis has been studied in pediatric patients with other immunosuppressive conditions, little is known about the efficacy and necessity of antibiotic prophylaxis in VA patients receiving sirolimus treatment.

The aim of this study was to determine the efficacy of extended administration of TMP-SMZ in preventing infections in VA patients receiving oral sirolimus.

## Patients and methods

### Study design

We performed a retrospective, multicenter, observational study. All consecutive VA patients receiving oral sirolimus from August 1, 2013, to January 1, 2021, were eligible. Five tertiary referral centers collaborated in this study. Each participating site collected the clinical data. The principal investigation site at West China Hospital of Sichuan University analyzed the data. The institutional review board of each participating site approved this study. Written informed consent was obtained for the use of the data from the patients’ parents.

### Study population

The patient entry criteria included the following: (1) patients had a clinical diagnosis of VA; (2) the patients’ ages were 0–14 years; (3) sirolimus was administered for treatment of the VA; and (4) the patients must not have had an active infection during the 1 week prior to initiation of sirolimus treatment. According to the criteria published previously, the diagnosis of VA was based on clinical features and/or histological findings [11, 15]. The exclusion criteria were as follows: (1) patients who received any concomitant prophylactic antibiotics other than TMP-SMZ during sirolimus treatment and (2) patients who received any concomitant medical therapy for VAs (e.g., corticosteroids).

Medical records and/or databases were reviewed within each participating site: sex; age; VA location; VA type; complication of VA; treatment before starting sirolimus; and details of sirolimus administration, including age when first prescribed, dose and duration of treatment, adverse events; and duration of follow-up. The disease severity at baseline was scored by site investigators on a scale from 4 to 1 as previously described [15]. Some patients had been included in previous studies [9, 11, 15, 19].

### Study intervention

All patients were assigned to receive at least 12 months of sirolimus treatment. The starting dose of oral sirolimus was 0.8 mg/m^2^ administered orally twice daily, and subsequently titrated to achieve a trough blood level of 10–15 ng/mL. This blood level was chosen based on previous experience [15, 16]. Deceasing sirolimus target ranges were allowed for any grade 3 and 4 adverse effects. Any sirolimus dose reductions, cessations, or interruptions were recorded.

With the aim of preventing potential infections, the authors altered the management after February 2017. Oral TMP-SMZ (10 mg/kg b.i.d., t.i.w.) was prescribed for all patients at least during the first 12 months of sirolimus treatment. We chose the TMP-SMZ regimen because of its broad antimicrobial coverage (including methicillin-resistant *Staphylococcus aureus* strains and PJP), ease of administration, cost-effectiveness, and relative safety with extended use in pediatric populations [20, 21].

### Study outcomes

Study visits were scheduled at baseline and at 1, 2, 4, 8, 12, 24 and 36 weeks, and 12 months, or as clinically indicated. Adverse events were collected by site investigators and graded according to Common Terminology Criteria for Adverse Events, version 4.0 (CTCAE v4.0). Serious adverse events were defined as any grade ≥ 3 toxicities identified during the initial 12 months of treatment. The primary outcome was the percentage of patients with at least one serious infection during the initial 12 months of treatment. Secondary outcome measures included the incidence of serious infection, the incidence of all adverse events, and tolerability during the initial 12 months of treatment.

### Statistical analysis

Given that TMP-SMZ would be effective in reducing severe infections, with a primary outcome of 5% versus 10% for non-TMP-SMZ treatment. With 86 subjects per treatment group, we had 80% power to detect a difference of 5% between TMP-SMZ or non-TMP-SMZ treatment for the primary endpoint. Study outcome variable comparisons were performed with a Fisher’s exact test, chi-square test or Mann–Whitney *U* test.

Comparisons of baseline characteristics were constructed with the nonparametric Mann–Whitney *U* test for continuous variables and Fisher’s exact test or a chi-square test for categorical variables. Statistical analyses were conducted using SPSS 23.0 for Windows (SPSS Inc., Chicago, IL, USA). *P* values less than 0.05 were considered statistically significant.

## Results

Table 1 shows the detailed characteristics of the 307 subjects treated with sirolimus. In total, 195 patients received TMP-SMZ treatment, and the remaining 112 patients did not receive any antibiotic prophylaxis. The female: male ratio was 1.0: 1.2. The median age at sirolimus initiation was 5.0 years (interquartile range, 1.8–8.0 years). In total, 121 patients had kaposiform hemangioendothelioma (KHE). Other common VAs included venous malformation, lymphatic malformation and combined VA (Additional file 1: Table S1). The lower extremities were the most common anatomic locations of VA lesions. The most common therapy indications were pain, impaired function or other symptoms (e.g., bleeding) and life-threatening or potentially life-threatening conditions (e.g., KMP). Previous treatments mainly included medical therapies (101 patients [32.9%]), partial resection (78 patients [25.4%]), sclerotherapy (62 patients [20.2%]) and supportive care treatments (69 patients [22.5%]) (Additional file 1: Table S2).

**Table 1:** Demographic and clinical characteristics at baseline

Characteristics	Non-TMP-SMZ group n = 112	TMP-SMZ group n = 195	Total n = 307	*P* values
Sex, no. (%)				0.876^c^
Male	61 (54.5)	108 (55.4)	169 (55.0)	
Female	51 (45.5)	87 (44.6)	138 (45.0)	
Age (y)				0.820^d^
Mean (range)	5.1 (0.1–14.0)	5.0 (0.1–14.0)	5.1 (0.1–14.0)	
Median (IQR)	5.0 (1.5–7.8)	5.0 (2.0–8.0)	5.0 (1.8–8.0)	
Weight (kg)				0.749^d^
Mean (range)	18.4 (3.6–45.0)	18.4 (3.5–50.0)	18.4 (3.5–50.0)	
Median (IQR)	18.0 (11.1–24.0)	18.0 (11.6–24.0)	18.0 (11.5–24.0)	
Topography				1.000^e^
Head and neck	23 (20.5)	41 (21.0)	64 (20.8)	
Limbs	61 (54.5)	106 (54.4)	167 (54.4)	
Upper	22 (19.6)	37 (19.0)	59 (19.2)	
Lower	39 (34.8)	69 (35.4)	108 (35.2)	
Trunk	28 (25.0)	48 (24.6)	76 (24.8)	
Diagnosis, no. (%)				0.982^e^
Vascular tumors	47 (42.0)	92 (47.2)	139 (45.3)	
Kaposiform hemangioendothelioma	40 (35.7)	81 (41.5)	121 (39.4)	
Tufted angioma	7 (6.3)	11 (5.6)	18 (5.9)	
Vascular malformations	65 (58.0)	103 (52.8)	168 (54.7)	
Venous malformation	22 (19.6)	35 (17.9)	57 (18.6)	
Arteriovenous malformation	1 (0.9)	5 (2.6)	6 (2.0)	
Lymphatic malformation	18 (16.1)	31 (15.9)	49 (16.0)	
Combined vascular malformation	16 (14.3)	22 (11.3)	38 (12.4)	
Vascular malformation associated with other anomalies	8 (7.1)	10 (5.1)	18 (5.9)	
Previous therapies, no. (%)^a^	81 (72.3)	152 (77.9)	233 (75.9)	0.217^e^
Partial resection	32 (28.6)	46 (23.6)	78 (25.4)	
Sclerotherapy	23 (20.5)	39 (20.0)	62 (20.2)	
Embolization	16 (14.3)	22 (11.3)	38 (12.4)	
Laser	8 (7.1)	15 (7.7)	23 (7.5)	
Medical therapies	41 (36.6)	60 (30.8)	101 (32.9)	
Propranolol	16 (14.3)	21 (18.8)	37 (12.1)	
Corticosteroids	26 (23.2)	37 (33.0)	63 (20.5)	
Vincristine	5 (4.5)	8 (4.1)	13 (4.2)	
Supportive care treatments^b^	29 (25.9)	40 (20.5)	69 (22.5)	
Disease activity				0.526^d^
Mean (range)	2.9 (1.0–4.0)	2.8 (1.0–4.0)	2.8 (1.0–4.0)	
Median (IQR)	3.0 (2.0–3.0)	3.0 (2.0–3.0)	3.0 (2.0–3.0)	

*TMP-SMZ* Trimethoprim-sulfamethoxazole, *y* year, *IQR* Interquartile range

^a^One patient may have received more than one treatment regimen

^b^Supportive care treatments included anti-coagulation, fresh frozen plasma, cryoprecipitate and packed red blood cells

^c^*P* value was calculated with chi-square test

^d^*P* value was calculated using the Mann–Whitney *U* test

^e^*P* value was calculated using the Pearson chi-square test

There were no significant differences in sex, age, type of VA or rate of combination treatment between the TMP-SMZ treatment group and the non-TMP-SMZ treatment group (Table 1). Baseline disease activity was similar between the two groups (*P* = 0.526). Over 95% of patients in each group enrolled in the study completed the initial 12 months of sirolimus treatment. The few patients who did not complete 12 months of treatment were mainly due to a lack of efficacy (3.6% in the TMP-SMZ treatment group vs. 4.4% in the nontreatment group; difference, 0.9%; 95% confidence interval (CI) − 4.0–7.4%).

In total, 35 (11.4%) subjects experienced 44 serious infections during the initial 12 months of treatment (Table 2). No significant between-group difference was observed in the percentage of patients with at least one serious infection (11.8% in the TMP-SMZ treatment group vs. 10.7% in the nontreatment group; difference, 1.1%; 95% CI − 7.0–8.0%). A potential causative agent was found in 35 serious infections, including 19 bacterial infections, 17 viral infections and 2 fungal infections (including 3 mixed infections). The most common serious infections were upper respiratory tract infection (in 7.2% of the patients in the TMP-SMZ treatment group and 6.3% of those in the nontreatment group; difference, 0.9%; 95% CI − 5.8–6.4%) and pneumonia (3.6% vs. 4.5%; difference, 0.9%; 95% CI − 3.6–6.7%). In addition, there was no significant difference in the overall incidence of adverse events between the TMP-SMZ treatment group and the nontreatment group (2.6 events per patient vs. 2.5 events per patient, *P* = 0.863) (Table 3). In both groups, no sirolimus-associated PJP or interstitial pneumonitis was noted.

**Table 2:** Serious infection reported during the initial 12 months of treatment

Safety outcomes^a^	Non-TMP-SMZ group n = 112	TMP-SMZ group n = 195	*P* values	OR (95% CI)
Percentage of patients with at least one serious infection, No. (%)^b^	12 (10.7)	23 (11.8)	0.774	0.897 (0.428–1.881)^d^
Serious infection, No. (%)				
Upper respiratory infection	7 (6.3)	14 (7.2)	0.756	0.862 (0.337–2.203)^d^
Pneumonia	5 (4.5)	7 (3.6)	0.703	1.255 (0.389–4.051)^d^
Cutaneous infection^c^	2 (1.8)	3 (1.5)	1.000	1.164 (0.191–7.071)^e^
Gastroenteritis	1 (0.9)	2 (1.0)	1.000	0.869 (0.078–9.697)^e^
Urinary tract infection	0 (0)	2 (1.0)	0.535	1.010 (0.996–1.025)^e^
Lymph gland infection	1 (0.9)	0 (0)	0.365	0.991 (0.974–1.009)^e^
Total	16	28	0.818^f^	N/A

*TMP-SMZ* Trimethoprim-sulfamethoxazole, *N/A* Data not available

^a^Adverse events were assessed with the Common Terminology Criteria for Adverse Events, version 4.0. A serious infection was defined as any of the grade ≥ 3 infections

^b^One patient may have had more than one serious infection

^c^Cutaneous infection included cellulitis, subcutaneous abscess and worsening skin ulceration

^d^The values were calculated using a chi-square test

^e^The values were calculated with Fisher’s exact test

^f^*P* value was calculated with nonparametric Mann–Whitney *U* test

**Table 3:** Total adverse events reported during the initial 12 months of treatment^a^

Category	Non-TMP-SMZ group n = 112	TMP-SMZ group n = 195	*P* values	OR (95% CI)
Mucositis	49 (43.8)	81 (41.5)	0.706	1.095 (0.648–1.751)^b^
Upper respiratory infection	30 (26.8)	51 (26.2)	0.904	0.033 (0.610–1.748)^b^
Nausea/vomiting	28 (25.0)	50 (25.6)	0.901	0.967 (0.566–1.651)^b^
Pneumonia^b^	26 (23.2)	48 (24.6)	0.782	0.926 (0.536–1.599)^b^
Thrombocytosis	22 (19.6)	37 (19.0)	0.886	1.044 (0.580–1.879)^b^
Cough	18 (16.1)	30 (15.4)	0.873	1.053 (0.557–1.991)^b^
Eczema	15 (13.4)	29 (14.9)	0.722	0.885 (0.452–1.733)^b^
Diarrhea	16 (14.3)	25 (12.8)	0.716	1.133 (0.577–2.227)^b^
Increased liver enzyme levels	11 (9.8)	22 (11.3)	0.691	0.856 (0.399–1.839)^b^
Constipation	12 (10.7)	20 (10.3)	0.899	1.050 (0.493–2.238)^b^
Rash	7 (6.3)	16 (8.2)	0.531	0.746 (0.297–1.872)^b^
Pain	6 (5.4)	12 (6.2)	0.775	0.863 (0.315–2.367)^b^
Gastroenteritis	6 (5.4)	10 (5.1)	0.931	1.047 (0.370–2.962)^b^
Cutaneous infection	5 (4.5)	11 (5.6)	0.655	0.782 (0.264–2.310)^b^
Acne	5 (4.5)	10 (5.1)	0.795	0.864 (0.288–2.596)^b^
Decreased appetite	5 (4.5)	9 (4.6)	0.951	0.966 (0.315–2.956)^b^
Lymphopenia	4 (3.6)	10 (5.1)	0.529	0.685 (0.210–2.238)^c^
Lymph gland infection	4 (3.6)	7 (3.6)	1.000	0.955 (0.285–3.476)^c^
Hyperlipidemia	3 (2.7)	8 (4.1)	0.752	0.643 (0.167–2.476)^c^
Hyperhidrosis	3 (2.7)	5 (2.6)	1.000	1.046 (0.245–4.461)^c^
Delay wound healing	2 (1.8)	5 (2.6)	1.000	0.691 (0.132–3.621)^c^
Hypercholesterolemia	2 (1.8)	3 (1.5)	1.000	1.164 (0.191–7.071)^c^
Urinary tract infection	2 (1.8)	3 (1.5)	1.000	1.164 (0.191–7.071)^c^
Alopecia	2 (1.8)	2 (1.0)	0.624	1.755 (0.244–12.630)^c^
Anemia	1 (0.9)	2 (1.0)	1.000	0.869 (0.078–9.697)^c^
Neutropenia	1 (0.9)	0 (0)	0.365	0.991 (0.974–1.009)^c^
Peripheral edema	0 (0)	1 (0.5)	1.000	1.005 (0.995–1.015)^c^
Total	285	507	0.863^d^	N/A

*TMP-SMZ* Trimethoprim-sulfamethoxazole, *N/A* Data not available

^a^Adverse events were assessed using the Common Terminology Criteria for Adverse Events, version 4.0

^b^The values were calculated using a chi-square test

^c^The values were calculated using a Fisher’s exact test

^d^*P* value was calculated using a nonparametric Mann–Whitney *U* test

Serious adverse events leading to discontinuation of sirolimus or target reduction of sirolimus, but not necessarily patient removal from the study, occurred in 26 (13.3%) patients receiving TMP-SMZ and 14 (12.5%) patients without TMP-SMZ (difference, 0.8%; 95% CI − 7.6–8.2%). The most frequent adverse event causing treatment discontinuation was pneumonia, followed by upper respiratory tract infections, mucositis and increased liver enzyme levels. No sirolimus-related deaths or permanent treatment discontinuations were reported throughout the study periods.

## Discussion

The off-label use of oral sirolimus in VA patients requires a thoughtful risk–benefit analysis and careful follow-up. Severe infections are a major concern during sirolimus treatment. Remarkably, there are infection-related deaths attributed to sirolimus in young patients with VA [18]. It is likely that many treating physicians would tentatively agree on the need to provide prophylactic antibiotics during sirolimus treatment. In the present study, we demonstrated an overall serious infection rate of 11.4% in VA patients receiving oral sirolimus treatment. In addition, we provided direct evidence that prophylactic use of TMP-SMZ neither reduced the infectious complication rate nor improved the tolerance to sirolimus monotherapy. Our data suggest that routine use of prophylactic antibiotics should not be mandated for all VA patients receiving sirolimus monotherapy.

The most concerning side effect of oral sirolimus in pediatric patients is immunosuppression, although there is evidence suggesting that immunologic parameters are hardly affected by sirolimus during 6 months of treatment in patients with Vas [22]. In a multicenter retrospective study reporting 113 patients treated with sirolimus (serum levels, 2.7–21 ng/ml), 17 severe adverse events were identified, including 8 viral pneumonias [23]. In a prospective multicenter study of sirolimus (target serum levels, 10–15 ng/ml) for complicated VAs, the most common adverse events and severe adverse events were mucositis and upper respiratory infection, respectively [11]. Similarly, in a recent randomized clinical trial (RCT) of 73 patients with KMP receiving either sirolimus or sirolimus (target serum levels, 10–15 ng/ml) plus prednisolone, the most common adverse events were upper respiratory tract infection and mucositis [9]. In another RCT including 59 children with a slow-flow vascular malformation receiving oral sirolimus (target serum levels, 4–12 ng/ml), the most frequent adverse event was an oral ulcer (49.2%) [10]. However, it is difficult to compare the adverse event rates among different studies. The large disparity in adverse event rates may be a testament to the broad heterogeneity of outcome definitions, confounding with other therapies (e.g., embolization, vincristine and corticosteroids), various durations of follow-up, and heterogeneity of sirolimus serum levels in these studies.

The risk of infection is highest in the early immunosuppressive therapy period and may be caused by bacterial, fungal, or viral pathogens [20]. One might consider that all immunosuppressed children should receive prophylaxis as the benefit may exceed the risk. In a systematic review by Freixo et al., antibiotic prophylaxis with TMP-SMZ was reported in 29.4% of patients with VA receiving sirolimus treatment. Infectious complications were reported in 2.5% and 5.2% of patients treated with TMP-SMZ and patients not treated with TMP-SMZ, respectively [12]. However, there are theoretical concerns regarding prophylaxis-induced antimicrobial resistance and hypersensitivity reactions [24, 25]. In the present study, we developed strategies that included TMP-SMZ prophylaxis, the aim of which is to protect patients during the early treatment period when immunosuppression is most intense. We found that prophylactic TMP-SMZ did not provide any benefit in preventing serious infection in VA patients receiving oral sirolimus. It is important to note that routine antibiotic prophylaxis did not decrease upper respiratory tract infection and pneumonia, both of which were also common severe infections in previous studies [11, 16].

Another opposition against TMP-SMZ-based prophylaxis is derived from the fact that the PJP rate in VA patients receiving sirolimus may be much lower than that in patients with other conditions [20, 21]. TMP-SMZ-based antibiotic prophylaxis is routinely employed to decrease the risk of PJP. In particular, TMP-SMZ has been shown to significantly reduce the incidence of PJP in patients with acquired immune deficiency syndrome [26]. In patients with rheumatic diseases receiving long-term high-dose glucocorticoids, the PJP incidence and mortality rates were found to be lower in the TMP-SMZ group than in the control group [27, 28]. There is evidence that up to 35% of patients with hematologic malignancies can develop PJP if they do not receive antibiotic prophylaxis [29]. However, in the current study, we did not observe any PJP in any patient, which was similar to the results reported in previous studies with a large sample size [9–11, 16, 23]. It is thus likely that PJP would be uncommon in patients with VA. Remarkably, an PJP incidence of at least 3.5% has been proposed as a cutoff point to justify prophylaxis [21, 30]. VA Patients receiving sirolimus might represent a unique subgroup of iatrogenically immunosuppressed subjects. Although these patients usually need prolonged use of sirolimus, they may have a lower risk of PJP than patients with other conditions needing iatrogenic immunosuppression. Therefore, it does not appear necessary to routinely include PJP prophylaxis (e.g., TMP-SMZ) in the management of VA patients receiving oral sirolimus.

Sirolimus trough levels in the blood should be monitored to avoid supratherapeutic levels and overimmunosuppression. In the present study, the sirolimus dose and blood level were uniformly monitored. Although the ability to measure sirolimus serum blood levels makes the treatment well controllable, no consensus has developed on how to monitor VA patients who are on sirolimus. There is also no consensus regarding the effective and safe sirolimus serum level for patients with VA. Ideally, sirolimus doses and target serum levels are based on the severity of VA, the goals of treatment, the individual patient and the treatment response. Interestingly, intermediate sirolimus doses (5–10 ng/ml) and low sirolimus doses (2–5 ng/ml) have been demonstrated to be efficacious in some patients with Vas [31, 32]. It is unknown whether the rate of serious infection can decrease with the presence of a low sirolimus dose. Clinically, high sirolimus doses may be needed in the initial treatment of patients with complicated VAs and patients with severe conditions (e.g., KMP), whereas low sirolimus doses can be used in patients who have already achieved a favorable response but need prolonged maintenance therapy [33].

In the present study, we excluded patients receiving a combined immunosuppressive regimen (e.g., the inclusion of corticosteroid), which may be associated with more significant immunosuppression than sirolimus monotherapy [34, 35]. Interestingly, previous studies revealed that the incidence of serious infection was not changed in KMP patients receiving sirolimus plus prednisolone [9]. This is expected given the relatively low dose of prednisolone and the short duration of combination treatment. In addition, all these patients received concomitant TMP-SMZ administration [9]. Clinically, the risk/benefit of administering any prophylaxis at all should be assessed on an individual basis. Patients considered at high risk for infections (e.g., patients with malignant neoplasms or pulmonary fibrosis), patients had a history of repeated infections (possibly low immunoglobulin levels, or severely neutropenia and lymphopenia), patients receiving more intense therapies (e.g., high-dose glucocorticoids and chemotherapy) and patients with the prolonged use of multiple immunosuppressive therapies may benefit from prophylactic antibiotics [20, 26, 27]. In these scenarios, cases of infants with complicated lymphatic anomalies (e.g., Gorhan-Stout disease and primary intestinal lymphangiectasia) can be associated with severe hypogammaglobulinemia and hypoalbuminemia [36]. It is those ‘high-risk’ patients who would theoretically be best served by extended TMP-SMZ therapy, and perhaps prophylactic antibiotics were helpful in preventing such patients from eventually developing a serious infection (e.g., PJP).

## Limitations

This study has several limitations due to its retrospective nature. First, the two groups were in different time periods during this study. Second, VAs are a heterogeneous group of diseases with different levels of severity. The broad clinical manifestations of the patients described in this study are therefore a limitation for statistical inference of safety outcomes. Third, the identification of adverse events was based on medical records and database reviews. Although we used centralized analyses and regular follow-up data, there is a potential for underestimation. Fourth, the advisability of prophylactic antibiotics remains unanswered because a significant difference in prophylactic effect between TMP-SMZ and other prophylactic antibiotics (e.g., a quinolone) has been revealed in patients with other conditions [37]. The relative sensitivity and specificity of the TMP-SMZ regimen compared with those of other regimens cannot be determined from the current study. However, prophylaxis with TMP-SMZ in children is relatively safe and well established. Finally, the present study was performed at several tertiary and quaternary care centers. This study design may have introduced a referral bias because of increased VA severity, and more aggressive therapies (higher sirolimus doses) than those in community practice may have been used.

## Conclusions

We conclude that prophylactic antibacterial antibiotics (e.g., TMP-SMZ) may not be mandated for all VA patients receiving oral sirolimus. Although our study did not reveal a need for routine antibacterial prophylaxis in VA patients receiving sirolimus, close monitoring of potential severe adverse events is needed in all patients. Due to the limitation of the research methodologies used, further RCTs are needed to establish and verify the prophylactic effect of TMP-SMZ in VA patients receiving sirolimus treatment.

## Supplementary Information

**Additional file 1: Table S1.** Detailed information about diagnosis at baseline. **Table S2.** Detailed information about previous therapies at baseline.

## Data Availability

The datasets used or analysed during the current study are available from the corresponding author on reasonable request.
