# Supplementary material for: Oral antibiotic prophylaxis for infection in patients with vascular anomalies receiving sirolimus treatment: a multicenter retrospective study
Source: Orphanet J Rare Dis. 2023 May 24;18:121. doi: 10.1186/s13023-023-02740-3 (PMC10207744; doi:10.1186/s13023-023-02740-3)
Supplement: Supplementary file 1 — Additional file 1: Table S1. Detailed information about diagnosis at baseline. Table S2. Detailed information about previous therapies at baseline. [file 13023_2023_2740_MOESM1_ESM.docx]

**Supplemental table I** **Detailed information about diagnosis at baseline**^a^

| Characteristics | non-TMP-SMZ group  n= 112 | TMP-SMZ group  n= 195 | Total  n= 307 | *P*-values |
| --- | --- | --- | --- | --- |
| Diagnosis, no. (%) |  |  |  | 0.982^b^ |
| Vascular tumors | 47 (42.0) | 92 (47.2) | 139 (45.3) |  |
| Kaposiform hemangioendothelioma | 40 (35.7) | 81 (41.5) | 121 (39.4) |  |
| Tufted angioma | 7 (6.3) | 11 (5.6) | 18 (5.9) |  |
| Vascular malformations | 65 (58.0) | 103 (52.8) | 168 (54.7) |  |
| Venous malformation | 22 (19.6) | 35 (17.9) | 57 (18.6) |  |
| Arteriovenous malformation | 1 (0.9) | 5 (2.6) | 6 (2.0) |  |
| Lymphatic malformation | 18 (16.1) | 31 (15.9) | 49 (16.0) |  |
| Combined vascular malformation | 16 (14.3) | 22 (11.3) | 38 (12.4) |  |
| Vascular malformation associated with other anomalies | 8 (7.1) | 10 (5.1) | 18 (5.9) |  |

^a^Abbreviation: TMP-SMZ, trimethoprim-sulfamethoxazole.

^b^*P* value was calculated using the Pearson chi-square test.

**Supplemental table II** **Detailed information about previous therapies at baseline^a^**

| Characteristics | non-TMP-SMZ group  n= 112 | TMP-SMZ group  n= 195 | Total  n= 307 | *P*-values |
| --- | --- | --- | --- | --- |
| Previous therapies, no. (%)^b^ | 81 (72.3) | 152 (77.9) | 233 (75.9) | 0.217^d^ |
| Partial resection | 32 (28.6) | 46 (23.6) | 78 (25.4) |  |
| Sclerotherapy | 23 (20.5) | 39 (20.0) | 62 (20.2) |  |
| Embolization | 16 (14.3) | 22 (11.3) | 38 (12.4) |  |
| Laser | 8 (7.1) | 15 (7.7) | 23 (7.5) |  |
| Medical therapies | 41 (36.6) | 60 (30.8) | 101 (32.9) |  |
| Propranolol | 16 (14.3) | 21 (18.8) | 37 (12.1) |  |
| Corticosteroids | 26 (23.2) | 37 (33.0) | 63 (20.5) |  |
| Vincristine | 5 (4.5) | 8 (4.1) | 13 (4.2) |  |
| Supportive care treatments^c^ | 29 (25.9) | 40 (20.5) | 69 (22.5) |  |

^a^Abbreviation: TMP-SMZ, trimethoprim-sulfamethoxazole.

^b^One patient may have received more than one treatment regimen.

^c^Supportive care treatments included anti-coagulation, fresh frozen plasma, cryoprecipitate and packed red blood cells.

^d^*P* value was calculated using the Pearson chi-square test.
